# Data for the synthesis of resorcinol–formaldehyde aerogels in acidic and basic media

**DOI:** 10.1016/j.dib.2017.04.029

**Published:** 2017-04-27

**Authors:** Daniel F. Molina-Campos, Rafael A. Fonseca-Correa, Diana P. Vargas-Delgadillo, Liliana Giraldo, Juan Carlos Moreno-Piraján

**Affiliations:** a Facultad de Ciencias, Departamento de, Grupo de Investigación en Materiales Porosos con Aplicaciones Ambientales, Universidad del Tolima, Colombia; b Facultad de Ciencias, Departamento de Química, Grupo de Investigación en Materiales Porosos y Aplicaciones Ambientales, Universidad del Tolima, Colombia; c Facultad de Ciencias, Departamento de Química, Universidad Nacional de Colombia, Bogotá, Colombia

**Keywords:** Aerogels. Isotherms, Immersion calorimetry

## Abstract

The aim of this research is to synthesise carbon aerogels and to compare the differences in their textural, morphological and chemical properties when synthesised in basic and acidic media, and with two different types of pretreatment carbonization and activation with CO_2_. Four samples are prepared and characterised using TGA–DTA, SEM, DRX, isotherm determination of N_2_ adsorption–desorption at −196 °C and immersion calorimetry. The data for pore distribution are reported using non-local density functional theory and quenched solid density functional theory. Finally, with the immersion calorimetry data, the consistency between the results using this technique and those obtained using the nitrogen isotherms is analysed.

**Specifications Table**

**Table:** 

Subject area	Chemistry
More specific subject area	Materials science: Carbons aerogels
Type of data	Tables, images, text, graphs and figures
How data was acquired	TGA–DTA (Hitachi model 7200), SEM, nitrogen isotherm of N_2_ at 77 K (IQ2, Quantachrome Inc.), XRD (Rigaku RU-300) and immersion calorimeter (“home-made”)
Data format	Raw and analysed
Experimental factors	Answer: The aerogels were synthesized using two different types of catalyst, acid and basic, and two different types of pretreatment, carbonization and activation with CO_2_ to determine these effects on the properties of the materials obtained.
Experimental features	Four carbon aerogel samples are synthesised from Resorcinol-Formaldehyde [1], [2]. These aerogels are characterised by adsorption-desorption isotherms of N_2_ at −196 °C. Their textural properties are analysed and their PSD are evaluated using Non Local Density Functional Theory (NLDFT) and Quenched Solid Density Functional Theory (QSDFT) with IQ2 software. It was found that the aerogel of smaller area BET corresponds to that synthesized in acid medium with a value of 377 m^2^ g^−^^1^ and the one of greater BET is the aerogel synthesized in basic medium whose value was 4314 m^2^ g^−1^, presenting type IV and II isotherms, respectively. Immersion calorimetry is determined in water and benzene to determine the hydrophobicity of the prepared aerogels. The synthesis of the aerogels in acidic media generates hydrophobic samples, while those obtained in alkaline media are hydrophilic.
Data source location	Facultad de Ciencias, Departamento de Química, Universidad de los Andes, Bogotá, Colombia
Data accessibility	Data are provided in this article

**Value of the data**

• The acidity or basicity in the preparation of carbon aerogels allows for the scientific analysis of the routes to generate solids with different properties.
• The detailed characterisation, both textural and chemical, is very useful for analysing the possible applications of these materials.
• The data show that with slight changes in the synthesis, can be prepared carbon aerogels with different textures and properties.
• Immersion calorimetry is a technique that is useful for evaluating textural and surface chemistry.

## 1 Data

**Fig. 1:**
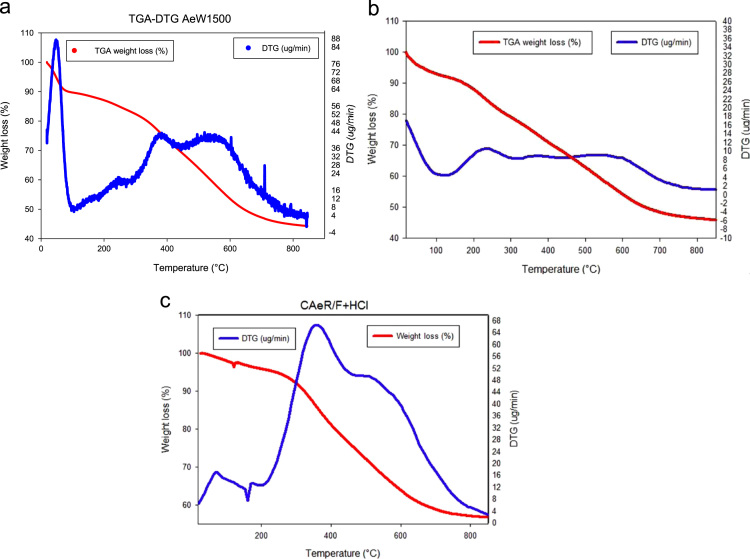
TGA–DTA of synthesised aerogels: a) AeW1500, b) AeR/F+HCl and c) CAeR/F+HCl.

**Fig. 2:**
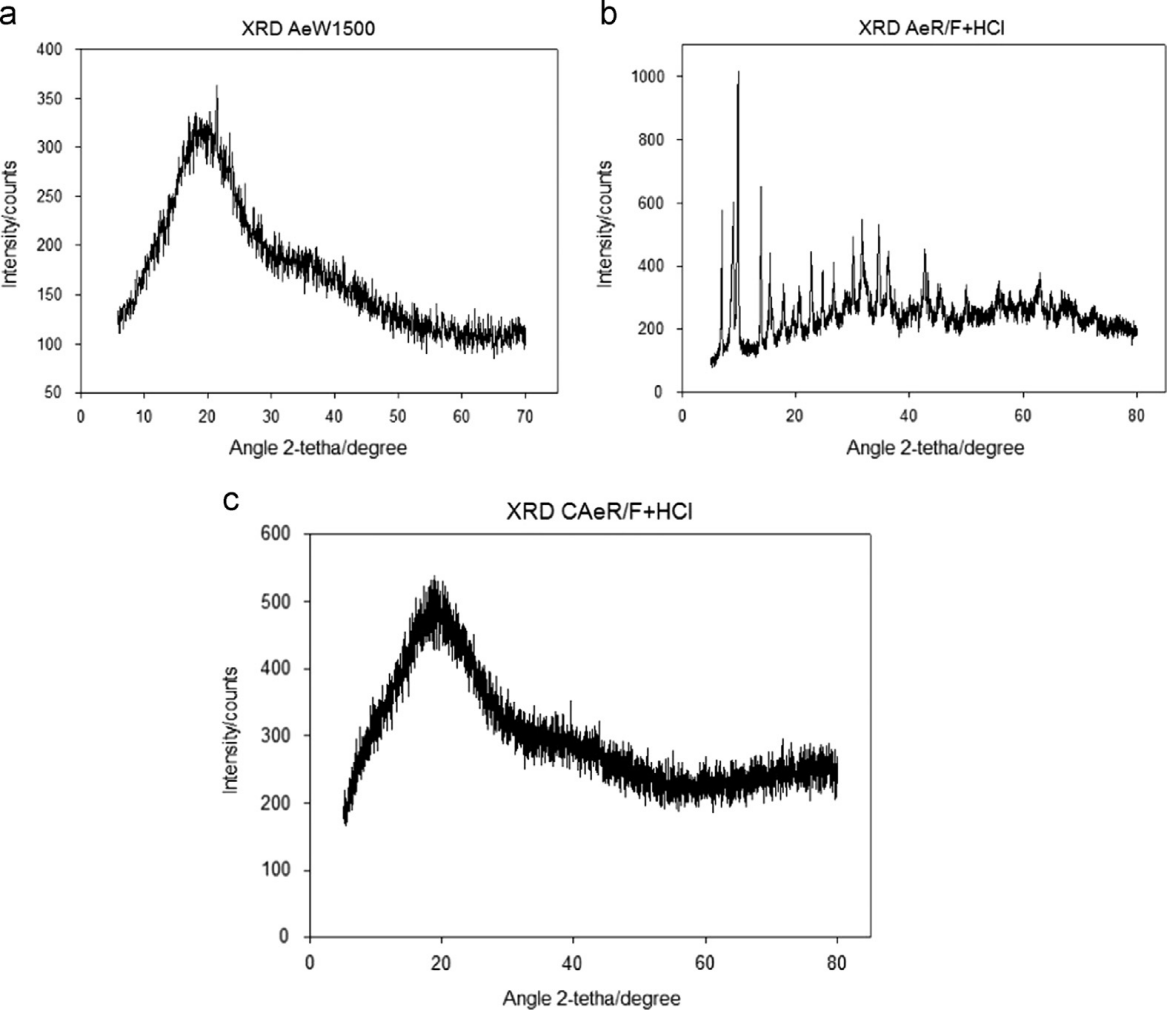
XRD of the carbon aerogels synthesised in this work: a) AeW1500, b) AeR/F+HCl and c) CAeR/F+HCl.

**Fig. 3:**
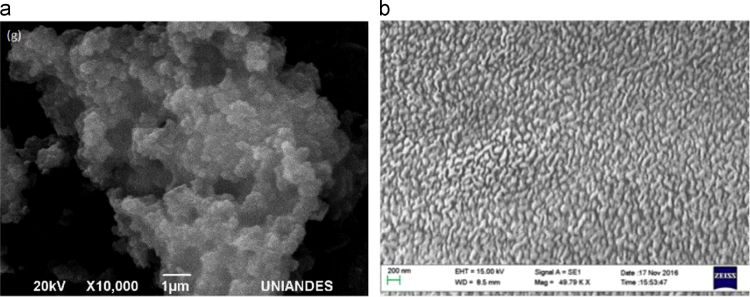
SEM images of a) AeW1500 and b) CAeR/F+HCl.

**Fig. 4:**
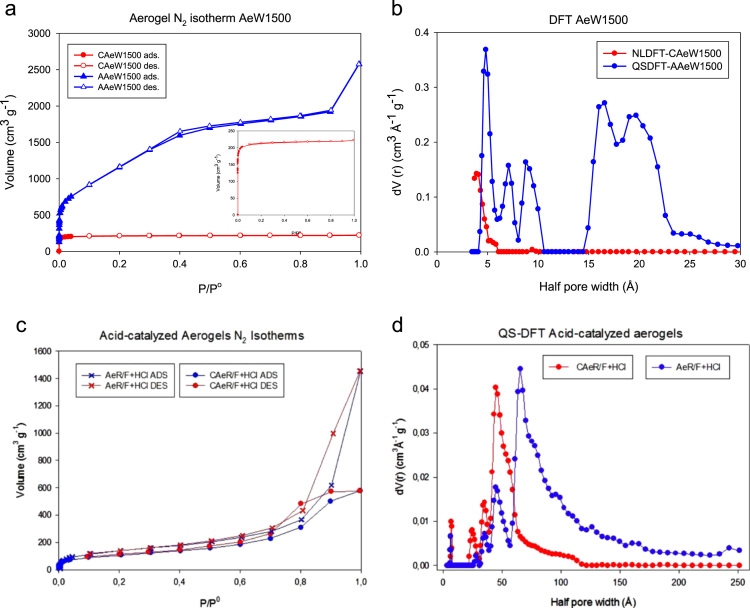
Adsorption isotherms of nitrogen on carbon aerogels at −196 °C and PSD QSDFT applied cylinder-slit model. a) Isotherms for AAeW1500 and CAeW1500, b) PSD applied NLDFT and QSDFT and cylinder-slit pore for AAeW1500 and CAeW1500, c) isotherms for AeR/F+HCl and CAeR/F+HCl, and d) PSD applied NLDFT and QSDFT and cylinder-slit pore for AeR/F+HCl and CAeR/F+HCl.

**Fig. 5:**
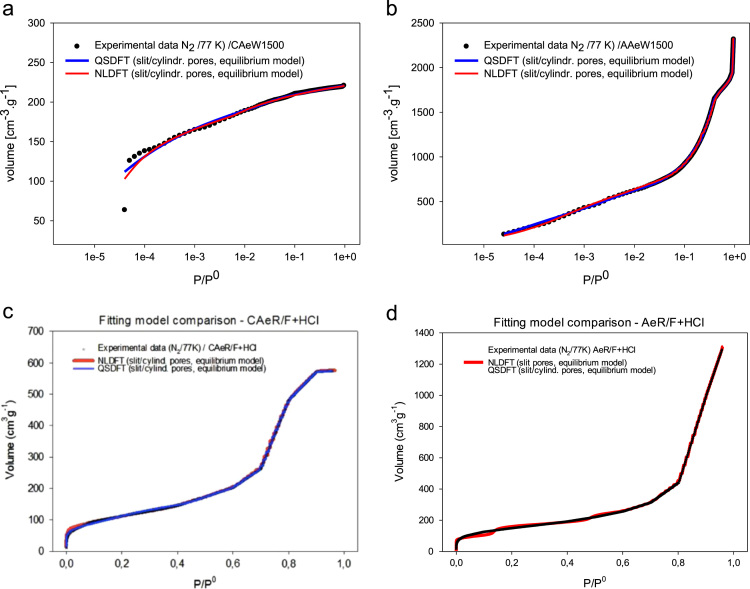
Comparison between QSDFT and NLDFT models for nitrogen adsorption of carbon aerogels. Experimental isotherms and fit from NLDFT and QSDFT plotted logarithmically (semi-scale) using a kernel cylindrical-slit for a) CAeW1500, b) AAeW1500, c) CAeR/F+HCl and d) AeR/F+HCl.

**Fig. 6:**
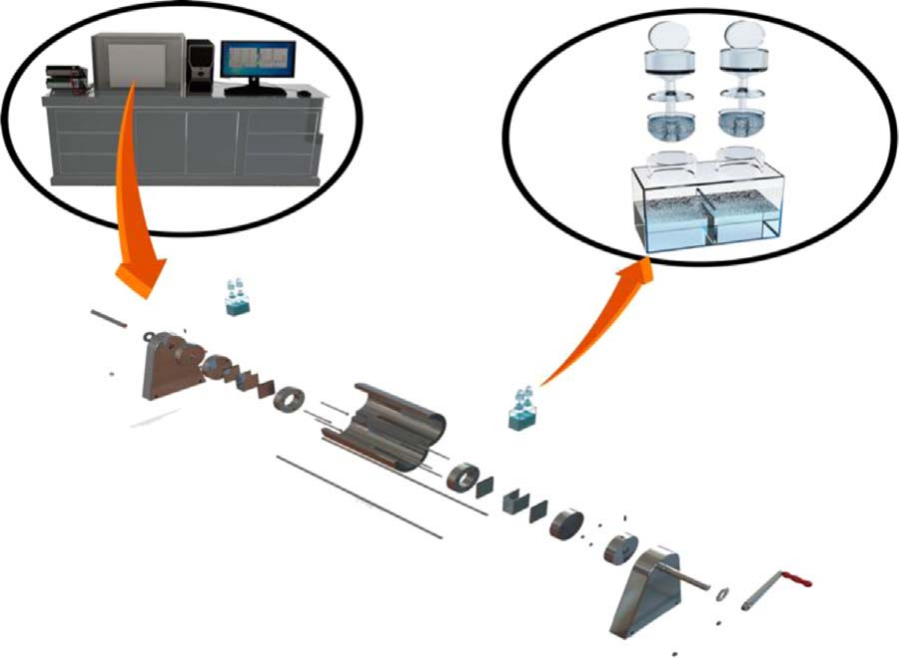
Immersion calorimeter used in this research [11], [12].

**Fig. 7:**
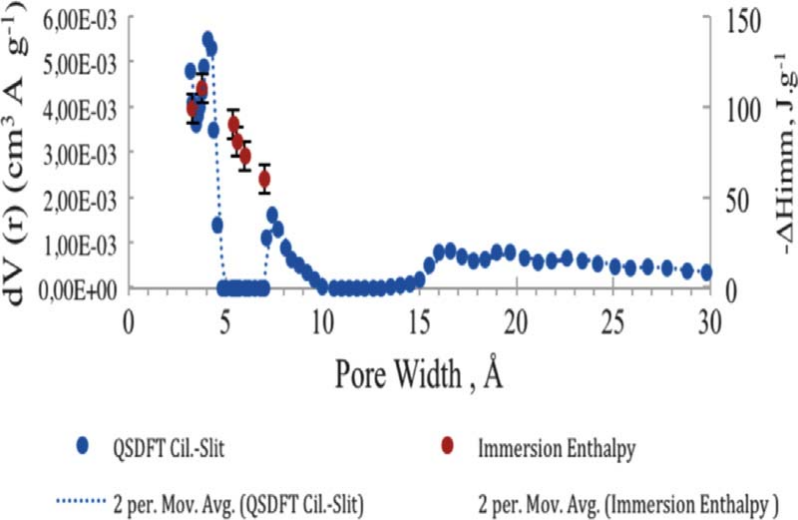
Comparison between the PSD calculated from the QSDFT kernel (from the isotherms of N_2_ to −196 °C) using cylindric-slit pores and the immersion enthalpy [13].

The Thermogravimetric Analysis–Differential thermogravimetric Analysis (TGA–DTA) of the aerogels is presented in Fig. 1. In Fig. 2, the X-ray Diffraction (XRD) images are presented. In Fig. 3 Scanning Electron Microscope (SEM) show images of aerogels synthesized. Fig. 4, the adsorption–desorption isotherms of N_2_ at −196 °C are shown. Fig. 5 shows the pore size distribution (PSD) obtained by applying the NLDFT and QSDFT kernels. Fig. 6 shows a scheme of the immersion calorimeter used to perform the determinations. Fig. 7 shows the relationship between the enthalpies of immersion and the PSD. Table 1 presents the data corresponding to the textural parameters of the synthesised aerogels and includes the index of hydrophobicity. Table 2 presents the corresponding data obtained for the PSD using the NLDFT and QSDFT kernels.

## 2 Materials, methods and experimental design

### 2.1 Materials

The formaldehyde (37% w/w aqueous solution methanol stabilised) and acetone (anhydrous) used in this data article were R.A. purchased from Aldrich™. Acetonitrile (ACS reagent grade) and hydrochloric acid (12.1 N) were purchased from Fisher Scientific™.

### 2.2 Synthesis of organic aerogels

Two methods, widely reported in the literature, were used for the aerogel synthesis [1], [2]. The first being carbon aerogels synthesised by base catalysis using the method described by Liu et al. [1] with some slight modifications in the reagent quantities, using the method of polymerisation sol–gel between monomers of resorcinol and formaldehyde (R and F) with sodium carbonate as a catalytic converter (C). Furthermore, aerogels were synthesised by the method reported by Mulik et al. [2] with acid as the catalyst. Supercritical drying is performed at 41 °C and 120 bar pressure in CO_2_ atmosphere in a high-pressure reactor. The carbonization is carried out in a horizontal carbolite furnace in N_2_ atmosphere at a flow rate of 100 mL/min and at a heating rate of 5° C/min to 850 °C. These same conditions are used for the activation but changing the gas for CO_2_.

The samples were labelled as follows:

CAeW1500 (aerogel synthesised at R/C ratio of 1500, in basic medium obtained after supercritical drying and after carbonization with N_2_)
AeW1500 (aerogel synthesised at R/C ratio of 1500, in basic medium obtained after supercritical drying and after the activation with CO_2_)
AeR/F+HCl (aerogel synthesised at R/C ratio of 8.4, in acid medium obtained after supercritical drying without activation with CO_2_)
CAeR/F+HCl (aerogel synthesised at R/C ratio of 8.4, in acid medium obtained after supercritical drying and after the carbonization with N_2_)

### 2.3 Experimental design

In Fig. 1(a), the TGA–DTA of the carbon aerogel synthesised in a basic medium (AeW1500) is presented, whereas Fig. 1(b) and (c) show the TGA–DTA corresponding to aerogels of carbon synthesised in an acidic medium (AeR/F+HCl and CAeR/F+HCl). The AeW1500 aerogel presents a slight drop at around 56 °C, which shows a decomposition at low temperature. Then, between the range of 100 to 400 °C, a strong mass change occurs. In the case of AeR/F+HCl, it has a drop at around 50 °C and then 200 °C uniformly drops up to 700 °C, while for CAeR/F+HCl has a mass loss of up to 300 °C and then presents a marked loss of mass between 200 and 600 °C, and is then stabilised after of 600 °C.

Fig. 2 shows the effect of the carbonization process on the aerogels samples after supercritical drying. In Fig. 1b shows the XRD for the AeR/F+HCl sample where the development of a crystalline structure is observed that is lost during the carbonization process, generating an amorphous structure as shown in Fig. 1c CAeR/F+HCl, and is corroborated with the XRD of the charred and activated sample AeW1500.

From the SEM images, it is possible to observe the difference of developed pores that are generated due to the two different mechanisms used to synthesise the carbon aerogels (see Fig. 3).

Fig. 4 shows the adsorption–desorption isotherms of N_2_ at −196 °C and the PSD obtained by applying the NLDFT and QSDFT kernels [3], [4], [5]. Fig. 4(a) and (b) show the data obtained for the AAeW1500 and CAeW1500 aerogels. AAew1500 has the isotherm type Ia according to the recent recommendations of the IUPAC [3], while the isotherm for CAeW1500 changes and becomes type II. The data obtained for the aerogels synthesised in an acidic medium are presented in Fig. 4(c) and (d). The isotherms for both the AeR/F+HCl and CAeR/F+HCl aerogels are type IVa [6] and have a hysteresis loop.

A comparative study of the experimental data according to NLDFT and QSDFT is presented in Fig. 5 by applying the cylindrical-pore size model. The QSDFT kernel introduces a rough surface as an additional structural parameter that characterises the heterogeneity of the wall pores. This model was applied to the samples synthesised in this research for carbon aerogels and allows us to obtain a more reliable pore size distribution [3], [4], [5]. Alternatively, NLDFT is based on having a smooth and homogeneous surface. The adjustments show that using the QSDFT algorithm and applying the cylinder-slit model is the best fit for AAeW1500, while NLDFT is the best fit for the CAeW1500 aerogel, in good agreement with the results obtained experimentally with the N_2_ isotherms at −196 °C. It is clear that according to the route of synthesis followed to obtain the carbon aerogels, rough and heterogeneous materials or with a smooth and homogeneous surface can be obtained. It is clear that the differences in the structures of the synthesized materials depends mainly on the synthesis processes in which the basic catalyst generates a more developed porous structure than with the acid catalyst since the formation of the enolate ion is greater in the first case which favors the formation of the monomer for the subsequent polymerization process, in the same way the activation process greatly increases the surface area compared to the untreated and carbonized samples.

Table 1, Table 2 present the data corresponding to the textural parameters of the aerogels prepared in this research. From the data published in Table 1, the micropore surface of the AAeW1500 sample reached a value of 4007 m g^−1^, a value that exceeds those reported in the literature [1]. Additionally, the hydrophobicity index is shown for each of the samples. For the aerogels catalysed in a basic medium, the HI obtained from the enthalpy ratio of immersion in water and in benzene, show that these are of hydrophobic character. While HI for the acid-catalysed aerogels have hydrophilic HI values.

Table 2 shows parameters that are deduced by applying the Dubinin–Astakhov (DA) [7] and Barrett–Joyner–Halenda (BJH) [8] models. A large BET surface area difference (Brunauer, Emmett, and Teller) [9], [10] lies between the CAeW1500 and AAeW1500 aerogels. For the latter, a BET area of 4314 m^2^ g^−1^ is obtained, which shows the effect of the treatment and the synthesis route on the specific BET area. Table 2 also shows the volume of mesopores (*V*_mes_) calculated from the BJH method [7]. The results for the aerogels of carbon synthesised in an acidic medium are also presented. Table 1, Table 2 show all the textural parameters of the synthesized samples, where the effect of the activation is demonstrated when the microporosity is developed, the mesoporosity values in the samples, that have not been treated and the values of pore volumes gives an indication of the porous structure of the samples. Adsorption energies are also observed, which is an important parameter for determining the affinity of the adsorption of the samples, and the hydrophobicity index that correlate quite well with the textural data.

Table 3 shows the values applying the NLDFT and QSDFT methods for all samples but using three different kernels to determine which geometry describes best the porous structure of the samples such as: cylindrical geometry, slit and a combination of the two cylindrical-slit models and the criterion of the best applied model is the least error of the methods found [3], [4], [5]. The calculated errors expressed as percentages of these (E%) are also presented. The volumes found by the DA [7], BJH [8] and the NLDFT and QSDFT models allow us to analyse whether or not there is mesoporosity and microporosity. In Fig. 5(a)–(d) we observe the relationship of the methods including the experimental data but only comparing the data with the cylinder-slit kernel which was the smallest error for all the samples, and that is the one that best describes the structure of the samples obtained observing a good concordance in the data.

Fig. 5 shows a schematic of the immersion microcalorimeter constructed in our laboratory, which was used to perform immersion calorimetry measurements [11], [12].

Fig. 6 shows a comparison between the PSDs calculated from the QSDFT kernel (shown as an example for the data obtained for a single sample of aerogel- AAeW1500-) and the enthalpies of immersion in molecules of different sizes following procedures suggested in the literature [13].

## Figures and Tables

**Table 1:** Textural parameters of aerogels with hydrophobicity index (HI).

	**Samples**	**Method D-R**					−**∆*H***_**imm**_**C**_**6**_**H**_**6**_**/**−**∆*H***_**imm**_**H**_**2**_**O**
		Average width of pore [Å]	Energy of adsorption [kJ mol^−1^]	*V*_micropore_	*S*_micropore_ [m^2^ g^−^^1^]	Correlation coefficient	HI
				[cm^3^ g^−^^1^]			
**Base-catalysed Aerogel**	CAeW1500	4.78	27.19	0.33	921	0.998	1.07
	AAeW1500	8.62	15.08	1.42	4007	0.998	1.17
**Acid-catalysed Aerogels**	AeR/F+HCl	8.99	14.44	0.17	479	0.999	0.61
	CAeR/F+HCl	8.96	14.51	0.12	346	0.991	2.57

**Table 2:** Analysis of S_BET_, DA and BJH methods.

	Samples	*S*_BET_ [m^2^ g^−1^]	D-A				BJH	
			*V*_micropore_ [cm^3^ g^−^^1^]	*E*_o_	*n*	Pore ratio [Å]	*V*_meso_ [cm^3^ g^−^^1^]	Pore ratio [Å]
				[kJ mol^−1^]				
**Base-catalysed Aerogel**	CAeW1500	858	0.334	9.17	1.8	6.6	0.01	18.2
	AAeW1500	4314	1.381	5.07	2.1	8.2	1.83	18.1
**Acid-catalysed Aerogels**	AeR/F+HCl	525	0.230	3.94	1.4	8.6	2.16	77.2
	CAeR/F+HCl	377	0.164	3.99	1.4	8.6	0.892	44.4

**Table 3:** Carbon aerogels: data report of PSD with NLDFT and QSDFT kernels and fitting error.

	Samples	Pore volume cyl [cm^3^ g^-1^]	Average width of pore cyl [Å]	*E* [%]	Pore volume cyl-slit [cm^3^ g^-1^]	Average width of pore cyl-slit [Å]	*E* [%]	Pore volume slit [cm^3^ g^-1^]	Average width of pore slit [Å]	*E* [%]
		NL-DFT								
**Base-catalysed Aerogel**	CAeW1500	0.34	5.85	1.41	0.33	3.92	1.07	0.31	4.30	2.73
	AAeW1500	3.52	17.8	1.25	3.50	8.90	1.25	3.41	13.8	2.18
		QS-DFT								
	CAeW1500	0.32	5.71	1.17	0.32	5.71	1.14	0.31	3.07	1.26
	AAeW1500	3.52	19.0	0.82	3.51	4.83	0.36	3.47	13.0	0.71
		NL-DFT								
**Acid-catalysed Aerogels**	AeR/F+HCl	1.98	74.9	2.28	1.98	74.9	2.28	1.99	44.6	1.84
	CAeR/F+HCl	0.882	47.2	1.03	0.88	47.2	0,971	0.861	35.6	2.17
		QS-DFT								
	AeR/F+HCl	2.02	65.6	1.20	2.02	65.6	1.18	1.98	44.9	1.80
	CAeR/F+HCl	0.87	45.0	1.24	0.87	45.0	1.20	0.87	28.8	1.40
